# The complete chloroplast genome of the invasive fern *Lygodium microphyllum* (Cav.) R. Br.

**DOI:** 10.1080/23802359.2018.1483755

**Published:** 2018-07-10

**Authors:** Graham A. McCulloch, James P. Hereward, Ellen C. Lake, Melissa C. Smith, Matthew F. Purcell, Gimme H. Walter

**Affiliations:** a School of Biological Sciences, The University of Queensland, Brisbane, Australia;; b Department of Zoology, University of Otago, Dunedin, New Zealand;; c United States Department of Agriculture, Agricultural Research Service, Invasive Plant Research Laboratory, Fort Lauderdale, FL, USA;; d United States Department of Agriculture, Agricultural Research Service, Australian Biological Control Laboratory, Brisbane, Australia

**Keywords:** Chloroplast genome, environmental weed, *Lygodium microphyllum*, Old World climbing fern

## Abstract

The Old World climbing fern, *Lygodium microphyllum*, is a rapidly spreading environmental weed in Florida, United States. We reconstructed the complete chloroplast genome of *L. microphyllum* from Illumina whole-genome shotgun sequencing, and investigate the phylogenetic placement of this species within the Leptosporangiate ferns. The chloroplast genome is 158,891 bp and contains 87 protein-coding genes, four rRNA genes, and 27 tRNA genes. Thirty-three genes contained internal stop codons, a common feature in Leptosporangiate fern genomes. The *L. microphyllum* genome has been deposited in GenBank under accession number MG761729.

The Old World climbing fern, *Lygodium microphyllum* (Cav.) R. Br. (Pteridophyta: Lygodiaceae), is a fast-growing vining fern. It is native to Africa, Asia, and Australia, and has become naturalized in a number of regions, including south and central Florida (United States) (Pemberton and Ferriter 1998). It is especially damaging to the Everglades, where it forms thick mats across trees in the swamp, and contributes to fire hazard. Due to its significant ecological and economic impacts, it is the target of large-scale biological control programmes (see Goolsby et al. 2006; McCulloch et al. 2018). Here, we report the complete chloroplast sequence of this weed and investigate its phylogenetic placement within the Leptosporangiate ferns.

We assembled the complete chloroplast genome from a *L. microphyllum* specimen collected from Jacksonville, Florida (30.241421°N, –81.911946°W; voucher 2016JAX14, USDA-ARS Australian Biological Control Laboratory, Brisbane). DNA was extracted from leaf material using CTAB (Doyle 1987) followed by spin column purification (Ridley et al. 2016). A-sequencing library was constructed using the NebNext Ultra DNA kit. Sequencing was conducted at Novogene (Beijing, China) on the Illumina HiSeq 2500 platform, yielding 80 million paired-end 150-bp sequences. Sequences were iteratively mapped to the *L. japonicum* chloroplast genome (KC536645) in Geneious v11.0.3 (Kearse et al. 2012), and some de-novo assembly of chloroplast reads was performed to correct insertions and deletions. Gene annotations were made through comparison to *L. japonicum*, and manually checked and edited.

The complete chloroplast sequence of *L. microphyllum* is 158,891 bp, the second largest of 74 fern genomes sequenced to date. Gene content, G + C%, and gene order were similar to those of *L. japonicum* (Gao et al. 2013). The complete chloroplast genome contains 118 genes, including 87 protein-coding genes, four rRNA genes, and 27 tRNA genes. Six protein-coding genes, four rRNA genes, and five tRNA genes were duplicated (or partially duplicated) in the inverted repeat region. Thirty-three genes had internal stop codons and incorrect start codons, a common feature in the chloroplasts of Leptosporangiate ferns (see Wolf et al. 2004; Guo et al. 2015).

The phylogenetic placement of *L. microphyllum* within the Leptosporangiate ferns was assessed using Bayesian inference. Representative chloroplast genomes from six of the seven Leptosporangiate orders were downloaded from GenBank. In addition, three Eusporangiate ferns were downloaded and included as outgroups. Sequences were aligned using MAFFT (Katoh and Standley 2013) and non-coding regions were removed. A Bayesian phylogeny was constructed using MrBayes 3.2 (Huelsenbeck and Ronquist 2001) under the GTR + I + γ model of nucleotide substitution (Figure 1). Four MCMC chains were run for 10,000,000 generations, with trees sampled every 1000 generations. *Lygodium microphyllum* is included in a well-supported clade with other Schizaeoid ferns (order: Schizaeales), with this clade sister to a clade containing the tree ferns (order: Cyatheales), Heterosporous ferns (order: Salviniales), and Polypod ferns (order: Polypodiales), consistent with previous phylogenies of this group (Pryer et al. 2004; Lu et al. 2015). This chloroplast genome provides a valuable resource for further resolving the evolutionary relationship among *Lygodium* species.

**Figure 1.**
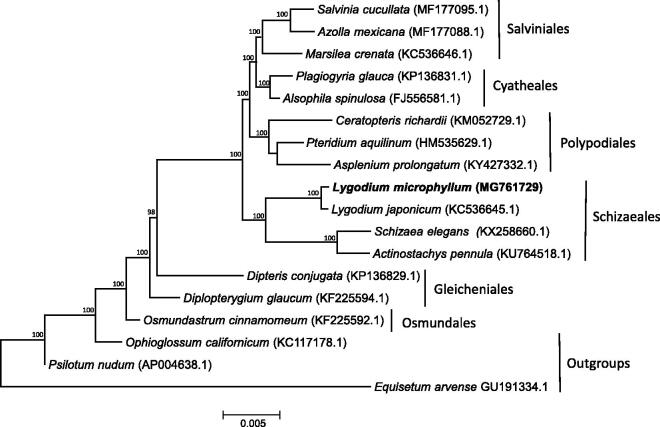
Bayesian maximum clade consensus phylogeny of Leptosporangiate chloroplast genomes. Posterior probabilities are noted above each note.
